# Notch Signaling Mediates Astrocyte Abnormality in Spinal Muscular Atrophy Model Systems

**DOI:** 10.1038/s41598-019-39788-w

**Published:** 2019-03-06

**Authors:** Kazuki Ohuchi, Michinori Funato, Yuta Yoshino, Shiori Ando, Satoshi Inagaki, Arisu Sato, Chizuru Kawase, Junko Seki, Toshio Saito, Hisahide Nishio, Shinsuke Nakamura, Masamitsu Shimazawa, Hideo Kaneko, Hideaki Hara

**Affiliations:** 1 Molecular Pharmacology, Department of Biofunctional Evaluation, Gifu Pharmaceutical University, Gifu, Japan; 2 Department of Clinical Research, National Hospital Organization, Nagara Medical Center, Gifu, Japan; 3 Department of Neurology, Toneyama National Hospital, Osaka, Japan; 4 Department of Occupational Therapy, Faculty of Rehabilitation, Kobe Gakuin University, Kobe, Japan

## Abstract

Spinal muscular atrophy (SMA) is an autosomal recessive neuromuscular disorder characterized by the degeneration of spinal motor neurons and muscle atrophy. The disease is mainly caused by low level of the survival motor neuron (SMN) protein, which is coded by two genes, namely *SMN1* and *SMN2*, but leads to selective spinal motor neuron degeneration when *SMN1* gene is deleted or mutated. Previous reports have shown that SMN-protein-deficient astrocytes are abnormally abundant in the spinal cords of SMA model mice. However, the mechanism of the SMN- deficient astrocyte abnormality remains unclear. The purpose of this study is to identify the cellular signaling pathways associated with the SMN-deficient astrocyte abnormality and propose a candidate therapy tool that modulates signaling. In the present study, we found that the astrocyte density was increased around the central canal of the spinal cord in a mouse SMA model and we identified the dysregulation of Notch signaling which is a known mechanism that regulates astrocyte differentiation and proliferation, in the spinal cord in both early and late stages of SMA pathogenesis. Moreover, pharmacological inhibition of Notch signaling improved the motor functional deficits in SMA model mice. These findings indicate that dysregulated Notch signaling may be an underlying cause of SMA pathology.

## Introduction

Spinal muscular atrophy (SMA) is an autosomal recessive disorder characterized by a loss of motor neurons, leading to skeletal muscle weakness and atrophy^1^. SMA cases are mainly caused by the deletions or mutations in *the* survival motor neuron 1 (*SMN1*) gene that is ubiquitously expressed, leading to the low level of SMN protein^2^. In SMA patients, an insufficient amount of SMN protein for motor neuron maintenance is produced by only *SMN2* gene which was nearly identical gene of *SMN1* gene^3^. However, it remains unclear why *SMN1* gene deletions or mutations specifically affects spinal motor neurons despite the ubiquitous expression of the gene. In previous reports, restoration of normal SMN protein levels in astrocytes, but not in motor neurons, extended the survival time of SMNΔ7 mice (*mSmn*^−/−^; *SMN2*^+/+^, *SMN2*Δ7^+/+^, null for the endogenous *Smn* gene and rescued with 2 human transgenes, *SMN2* and an *SMN2* ORF with exon 7 deleted), widely used as a model of severe SMA^4–9^. Several groups, including ours, have previously shown that glial fibrillary acidic protein (GFAP)- or S100-positive astrocytes are abundant in spinal motor neuron cultures derived from induced pluripotent stem cells (iPSC) obtained from SMA patients (SMA-iPSC MNs)^10,11^. Therefore, SMN-deficient astrocytes may be partially responsible for SMA pathogenesis. Some researchers indicated that the SMN-deficient astrocytes mediate the motor neuron alteration^12–14^.

Previous reports have proposed that astrocyte differentiation may be controlled by Notch signaling, a cell communication mechanism involving signal transmission between adjacent cells (juxtacrine signaling). This mechanism suppresses embryonic neurogenesis and promotes astrogenesis immediately after birth^15–18^. A recent SMA study showed that Notch signaling was dysregulated at late disease stages (postnatal day [PND] 11) in SMNΔ7 mice^19^.

Our recent report^11^ and Caraballo-Miralles *et al*.^19^ show that Notch signaling is dysregulated in the spinal cord of SMA model mice and astrocyte culture, while Maeda *et al*.^20^ demonstrated down-regulation of Notch signaling in embryonic stem cell derived motor neuron cultures. Based on the reports, the up- or down-regulation of Notch signaling was detected in different cell types.

In the present study, we focused on the upregulation of Notch signaling in SMN-deficient-astrocytes and examined the role of Notch signaling in abnormal GFAP-positive astrocytes in SMA model systems. Furthermore, we investigated whether pharmacological inhibition of Notch signaling rescues the motor function deficits in SMNΔ7 mice. Our results indicate that Notch signaling may be involved in SMA pathogenesis.

## Results

### GFAP-and S100-Positive Astrocyte Abnormalities in the Spinal Cords of SMNΔ7 Mice

**Figure 1:**
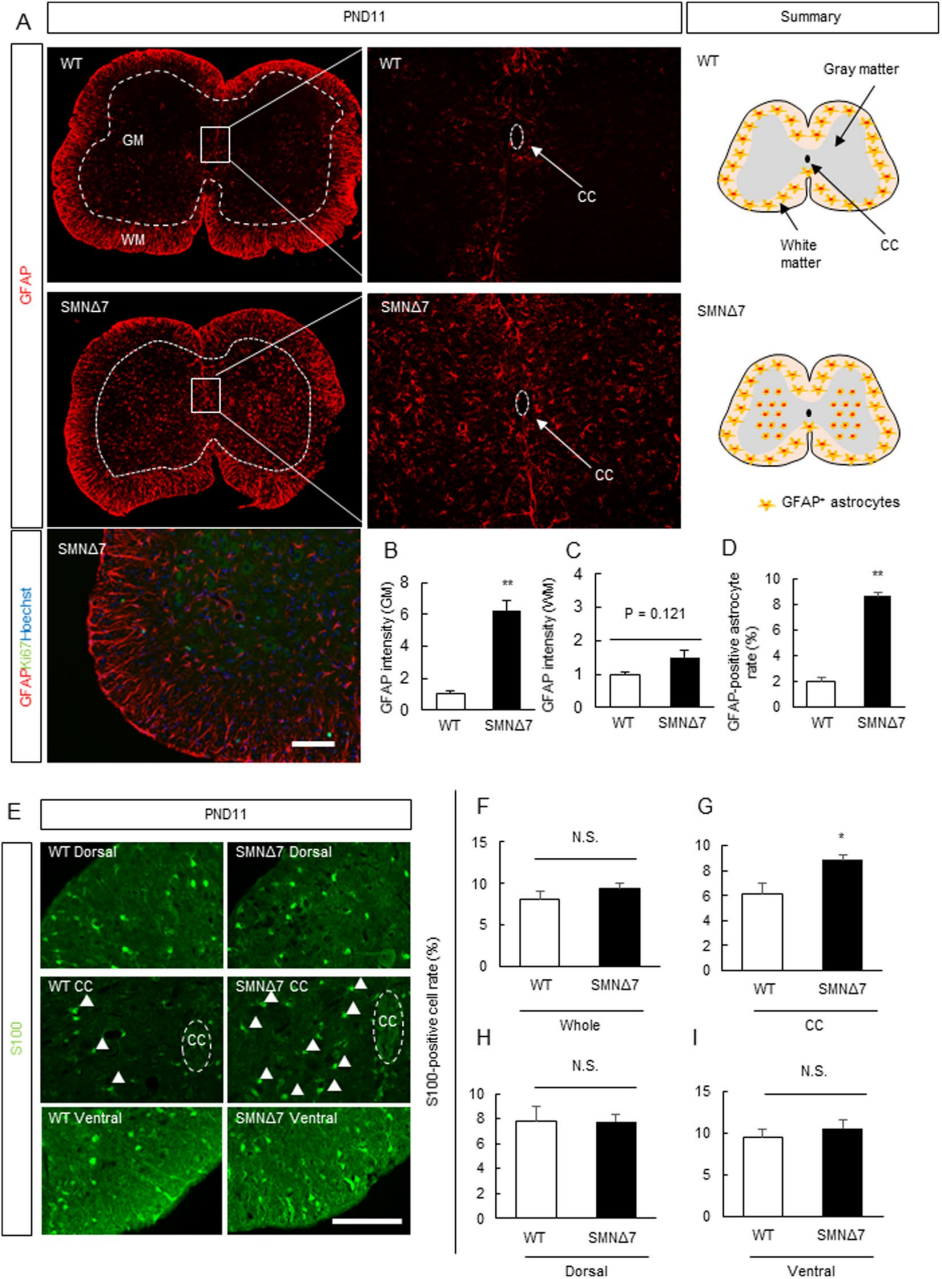
SMN-deficient astrocytes were abnormally increased in the spinal cord of SMNΔ7 mice. (**A**) GFAP-positive astrocytes were increased not in white matter (WM) but in the gray matter (GM) of spinal cord of WT (*mSmn*^+/+^, *SMN2*^+/+^, *SMN*Δ*7*^+/+^) and SMNΔ7 (*mSmn*^−/−^, *SMN2*^+/+^, *SMN*Δ*7*^+/+^) mice at PND11. In addition, Ki67-positive cells were not detected. Scale bar shows 200 μm. (**B,C**) Quantitative analysis of GFAP immunoreactivity in the GM and WM in the spinal cord of SMNΔ7 mice at PND11. Data are shown as means ± SEM. (n = 3 or 4). ^##^*p* < 0.01 versus WT (Student’s *t* test). (**D**) The quantitative analysis of GFAP-positive cells in the GM of the spinal cord of SMNΔ7 mice at PND11. Data are shown as means ± SEM. (n = 3 or 4). ^##^*p* < 0.01 versus WT mice (Student’s *t* test). (**E–I**) Quantitative analysis of S100-positive cells in the whole, ventral, dorsal and central canal in the spinal cord of WT and SMNΔ7 mice at PND11. Data are shown as means ± SEM. (n = 3 or 4). ^#^*p* < 0.05 versus WT mice (Student’s *t*-test). Scale Bar shows 100 µm.

Astrogliosis in the spinal cords of SMNΔ7 mice has been detected as increased GFAP immunoreactivity starting from PND5^9,19,21^; however, the pattern of astrogliosis in SMA has not been studied in depth. To elucidate the site of the astrocytic abnormality at the pathological stage (PND11), we investigated the expression pattern of GFAP (a marker of terminally differentiated astrocytes) in the lumbar spinal cord of SMNΔ7 mice, in which central nervous degeneration caused by ubiquitous SMN depletion preceded gastrocnemius atrophy (Supplemental Fig. 7A–F). GFAP immunoreactivity and GFAP-positive astrocytes were dramatically increased specifically in the gray matter (GM), which contains the cell bodies of numerous motor neurons, while remaining unchanged in the white matter (WM) (Fig. 1A–D). In addition, Ki67-positive proliferative cells were not detected in SMNΔ7 mice of PND11 (Fig. 1A).

Next, to determine whether the increase in GFAP immunoreactivity resulted from astrogliosis or astrogliogenesis, we measured the number of astrocytes positive for the other astrocyte marker, S100^10,14,22^. There was an increase in the number of S100-positive cells around the spinal cord central canal, but not in the spinal cord as a whole, or in its ventral and dorsal regions (Fig. 1E–I).

### Notch Intracellular Domain (NICD)-Signal Transducer and Activator of Transcription 3 (STAT3)-GFAP/S100 Axis is Dysregulated in the Spinal Cords of SMNΔ7 Mice and an *In Vitro* SMA Model

**Figure 2:**
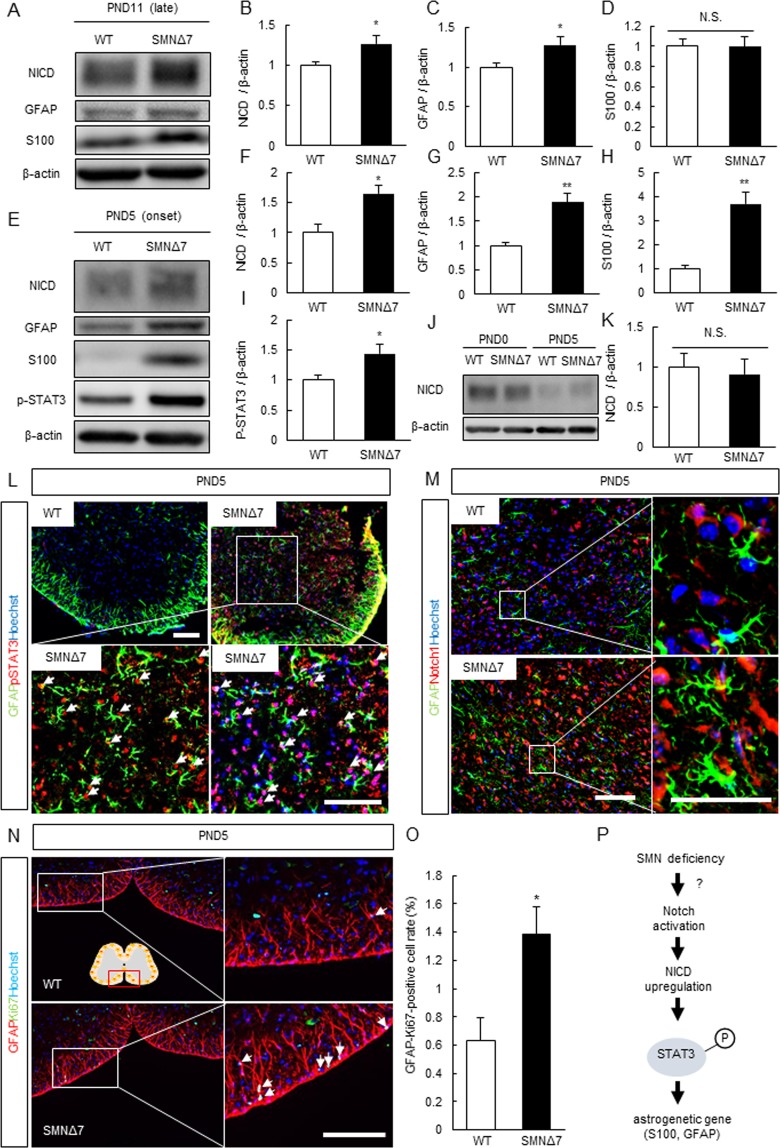
Notch signaling was dysregulated in the spinal cord of SMNΔ7 mice. (**A–D**) Notch signaling was dysregulated in the spinal cord of SMNΔ7 mice (PND11; late stage) by western blot analysis. Data are shown as means ± SEM. (n = 5 or 6), ^##^*p* < 0.01 and ^#^*p* < 0.05 versus WT mice (Student’s *t*-test). The cropped blots are used in this Figure and full-length blots are presented in Supplemental Fig. 1. (**E–I**) Expression of NICD, p-STAT3 and astrocytic marker including GFAP and S100 was increased in the spinal cord of SMNΔ7 mice (PND5; early stage) by western blot analysis (n = 3 or 4), ^##^*p* < 0.01, ^#^*p* < 0.05 versus WT mice (Student’s *t*-test). The cropped blots are used in this Figure and full-length blots are presented in Supplemental Fig. 2. (**J**,**K**) Expression of NICD did not change in the spinal cord of SMNΔ7 mice (PND0) by western blot analysis (n = 3). Data are shown as means ± SEM. (n = 3) (Student’s *t*-test). The cropped blots are used in this Figure and full-length blots are presented in Supplemental Fig. 3. (**L**) The representative fluorescence images of spinal cord in WT and SMNΔ7 mice (PND5). Almost all of GFAP-positive cells expressed the p-STAT3. Scale bar shows 100 µm. (**M**) The representative fluorescence images of spinal cord in WT and SMNΔ7 mice (PND5). Almost all of GFAP-positive cells expressed the Notch1. Scale bar shows 100 µm. (**N**) The representative fluorescence images of spinal cord in WT and SMNΔ7 mice (PND5). GFAP-Ki67-positive cell rate was increased in SMNΔ7 mice (PND5). Scale bar shows 100 µm. (**O**) The Quantitative analysis of GFAP-Ki67-positive cell rate in the spinal cord of WT and SMNΔ7 mice at PND5. Data are shown as means ± SEM. (n = 3). *p < 0.05 versus WT mice (Student’s *t*-test). (**P**) The Summary of Notch-STAT3-GFAP/S100 axis which may be dysregulated in SMA pathology.

Notch signaling has been shown to enhance astrogliogenesis in cooperation with STAT1/3^23,24^, with astrogliogenic genes such as GFAP and S100 dysregulated downstream of phosphorylated STAT3 (p-STAT3)^25^. Therefore, the Notch intracellular domain (NICD)-STAT3-GFAP/S100 axis plays an essential role in the astrogliogenesis in the developing central nervous system^26^. Thus, to elucidate the mechanism of the astrocytic abnormality in SMA pathology, we investigated the expression of astrogliogenic factors including NICD, p-STAT3, GFAP, and S100 by Western blot analysis. The Western blot data showed an upregulation of the NICD, p-STAT3, GFAP, and S100 levels in the spinal cords of SMNΔ7 mice at PND5 as well as the NICD, p-STAT3, and GFAP levels at PND11 (Fig. 2A–I). In contrast, S100 expression in the whole spinal cord was not changed at PND11 (Fig. 2D), consistent with the findings shown in Fig. 1E–I. To investigate the regulation of Notch signaling before the onset of SMA (PND5 in SMNΔ7 mice), we examined whether dysregulation of Notch signaling occurred on PND0 by Western blot analysis. The analysis showed that the expression of NICD did not change on PND0 and the expression level of NICD was lower in wild type (WT) mice on PND5 (Fig. 2J,K). Previously, it was reported that Notch1 is highly expressed in the brains of embryonic mice and humans, but its expression in the adult central nervous system is low^27,28^. Therefore, in SMA pathology, Notch signaling was not thought to be “activated” but “dysregulated”.

Next, to show whether Notch-STAT3 signaling is dysregulated specifically in astrocytes, we performed co-immunostaining of GFAP/Notch1 and GFAP/pSTAT3 using the spinal cords of WT and SMNΔ7 mice. According to our results, almost all GFAP-positive cells in spinal cord GM expressed pSTAT3 or Notch1, suggesting that Notch-pSTAT3 signal dysregulation (indicated by Western blot data) was caused specifically in astrocytes (Fig. 2L,M). Furthermore, to identify whether the SMN-deficient astrocytes are proliferative or not, we performed the co-immunostaining using anti-GFAP and Ki67 antibodies. Our results indicated that the ratio of GFAP-Ki67-positive cells /total cells significantly increased, suggesting that proliferative astrocytes (Ki67-positive cells surrounded by GFAP) increased in spinal cords of SMNΔ7 mice (Fig. 2N,O). Taken together, the increase in GFAP intensity was partly contributed to the astrocyte proliferation and the astrocytic abnormality in SMA is attributable to the dysregulation of the NICD-STAT3-GFAP/S100 axis (Fig. 2P).

Once Notch signaling is activated in a stem cell culture, neural stem cells become neural progenitor cells that send inhibitory signals to neural stem cells, resulting in a location pattern in which differentiated cell and neural stem cells are always adjacent to each other^29,30^. This short-range intercellular action specifically in Notch signaling is called lateral inhibition. To test if Notch signaling might be dysregulated at earlier stages of SMA, we investigated the expression patterns of NESTIN, a marker of neuronal stem cells, and NOTCH1 using both WT and SMA-iPSC culture. An SMA 01 line was already established in our previous report^11^. In the present study, to analyze the dysregulation of Notch signaling using 3 SMA patients, we established SMA-iPSC 06 and 07 lines. *SMN2* gene copy number was 3 copies in SMA-iPSC (SMA01), 2 copies in SMA-iPSC (SMA06) and 3 copies in SMA-iPSC (SMA07), respectively. *SMN2* gene copy number was analyzed by SALSA MLPA KIT P021-A1 SMA (MRC-Holland, Amsterdam, Holland). Consequently, we confirmed the presence of pluripotency markers in SMA06 and 07 lines, revealed an un differentiated phenotype and identified 3 germ layers (ectoderm, endoderm and mesoderm) in the 2 patient-derived iPSC lines (SMA06 and 07) by examining undifferentiated and layer-specific marker expression (Supplemental Fig. 8B,C).

**Figure 3:**
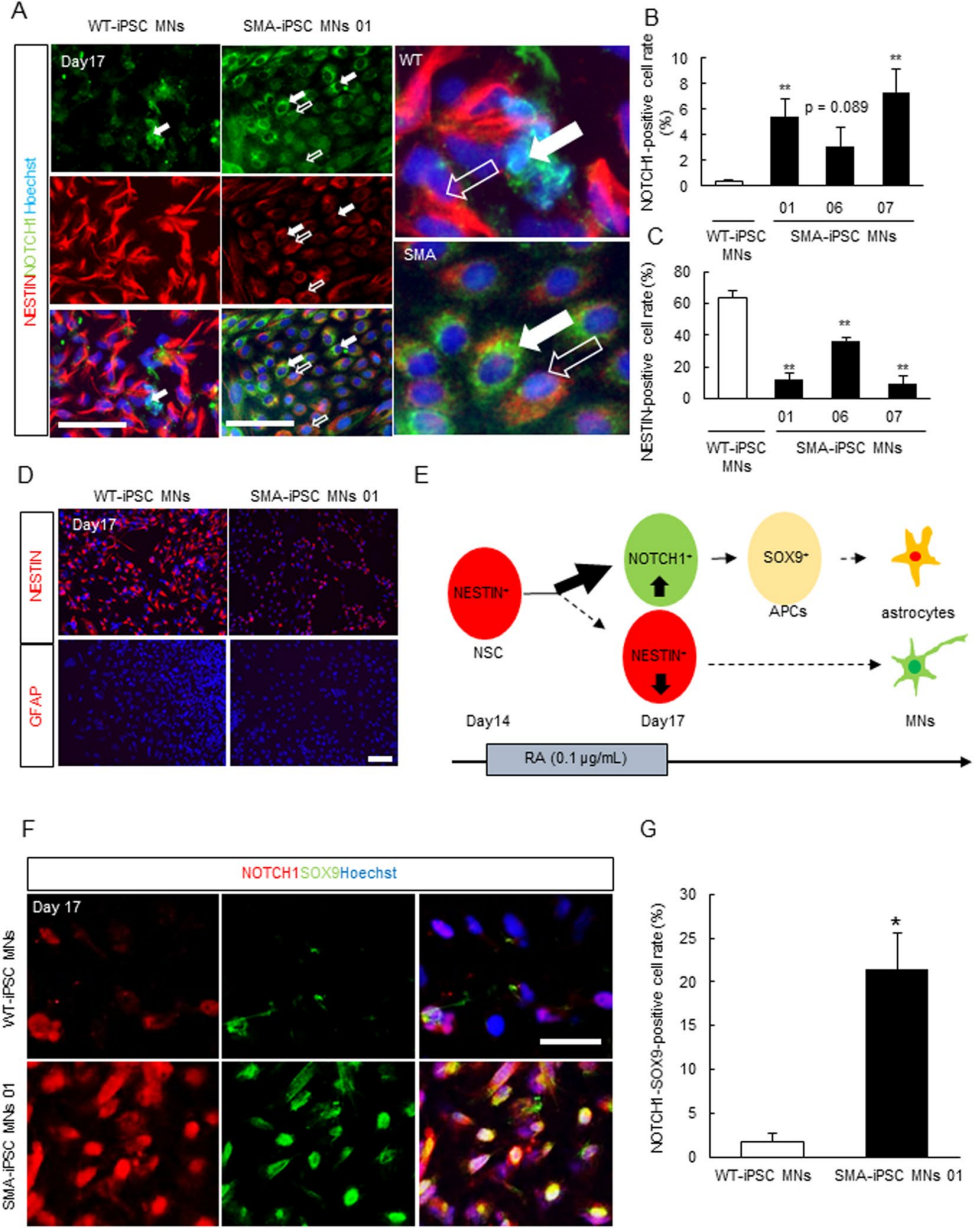
Notch signaling was dysregulated in SMA-iPSC culture system. (**A–C**) NOTCH1-positive cells (n = 3 or 4) were increased in SMA-iPSC culture and Nestin-positive cells which indicate neuronal stem cells (n = 3 or 4) was decreased in SMA-iPSC, Values represent the mean ± SEM. ^##^*p* < 0.01 and ^#^*p* < 0.05 versus WT-iPSC MNs (Student’s *t*-test). Scale bar shows 50 µm. (**D**) At day17 after motor neuron induction when Nestin-positive cells were abundant in WT-iPSC culture, GFAP which is expressed in also neuronal stem cell was not detected. Scale bar shows 50 µm. (**E**) The scheme of cell fate of NOTCH1^high^, SOX9-positive and Nestin^high^ cell. (**F**,**G**) SOX9-NOTCH1-positive cells were increased in SMA-iPSC culture (n = 3). Values represent the mean ± SEM. **p < 0.01 versus WT-iPSC culture (Student’s *t*-test). Scale bar shows 50 µm.

Previously, we confirmed the presence of an undifferentiated phenotype and 3 germ layers of SMA01 iPSC lines^11^. These SMA-iPSC lines in the present study exhibited normal karyotypes (Supplemental Fig. 8D). Retinoic acid (RA), which induces spinal motor neuron differentiation, was added to the culture media on days 14–17. At day 17, the Nestin expression level had decreased in SMA-iPSC culture compared to that in WT-iPSC culture when analyzed using a protocol for motor neuron induction (Fig. 3A,C,D; Supplemental Fig. 8A)^11^. Both NOTCH1 expression in the cell membrane (Fig. 3A,B), and the NICD levels in the nucleus (indicated by Hoechst 33342 staining a marker of both living and dead cells) had increased in SMA-iPSC culture by day 17 (Supplemental Fig. 9A,B). Interestingly, NOTCH1^high^ differentiated cells and NESTIN^high^ neural stem cells were always adjacent to each other in SMA-iPSC culture, (Fig. 3A, white arrows; NOTCH1^high^ cell and outline arrow; NESTIN^high^ cell).

Taken together, our results suggest that Notch signaling is dysregulated at earlier SMA stages, and likely drives the transition of NOTCH1-positive cells into immature astrocytes in SMA-iPSC culture, although GFAP-positive differentiated astrocytes were not detected (Fig. 3D). Furthermore, to verify that SMA-iPSC culture at day 17 when mature astrocytes have not emerged, contained immature astrocytes that express NOTCH1, we performed co-immunostaining using a combination of anti-NOTCH1 and anti-SOX9 antibodies in astrocyte precursor cells^31^. The data indicate that NOTCH1-SOX9-positive cells had increased in SMA-iPSC culture by day 17, suggesting that astrocyte differentiation may be promoted in SMA-iPSC culture (Fig. 3E–G).

### SMA-Patient-Derived iPSC Derived Culture Medium Including Abundant GFAP-Positive Astrocytes Induced Motor Neuron Death

**Figure 4:**
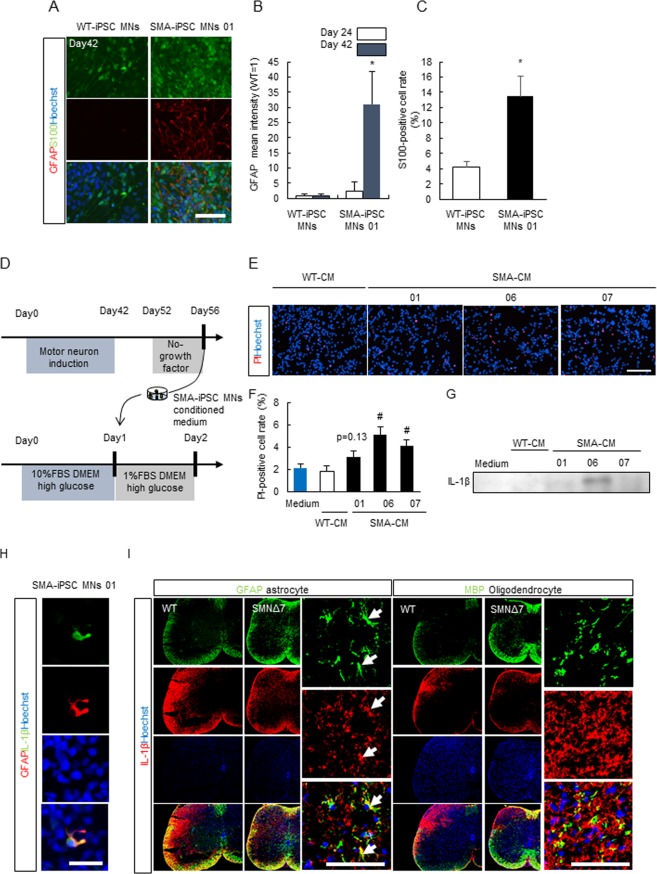
GFAP-positive astrocytes in SMA-iPSC culture induced motor neuronal cell death. (**A**) The representative fluorescence images of WT- and SMA-iPSC culture stained with GFAP and S100 at Day 42. Scale bar shows 50 μm. (**B**) The quantitative analysis of GFAP immunoreactivity in WT- and SMA-iPSC culture. GFAP immunoreactivity was increased in SMA-iPSC MNs (Day 24, n = 4; Day 42, n = 6 or 8). Values represent the mean ± SEM. *p < 0.05 versus WT-iPSC MNs (Student’s *t*-test). (**C**) The quantitative analysis of S100-positive cell rate in WT- and SMA-iPSC culture. S100-positive cells were increased in SMA-iPSC MNs. (Day 42, n = 3 or 4). Values represent the mean ± SEM. *p < 0.05 versus WT-iPSC MNs (Student’s *t*-test). (**D**) The protocol of cell death assay using conditional medium of SMA-iPSC MNs (SMA-CM). (**E**) The representative fluorescence images of NSC34 cells stained with Hoechst 33342 (blue) and propidium iodide (red) after 24 h WT- or SMA-CM treatment. Scale bar shows 50 μm. (**F**) The quantitative analysis of the ratio of PI-positive cells to Hoechst 33342-positive cells treated with or WT- or SMA-CM treatment. Each column and bar represents mean ± SEM (n = 6). ^##^*p* < 0.01 versus WT-CM (Student’s *t*-test). (**G**) Representative images of Western blot analysis of IL-1β expression in WT- or SMA-CM. The cropped blots are used in this Figure and full-length blots are presented in Supplemental Fig. 4. (**H**) The representative images of IL-1β/GFAP in SMA-iPSC MNs. IL-1β was localized in GFAP-positive cells. Scale bar shows 50 μm. (**I**) The representative images of IL-1β/GFAP and IL-1β/MBP using WT and SMNΔ7 mice. IL-1β was localized in GFAP-positive cells (astrocytes), not MBP-positive cells (oligodendrocytes). Scale bar shows 100 µm.

Spinal motor neurons are selectively affected in SMA despite SMN protein levels being depleted ubiquitously; the mechanism of the cell type specification remains unclear^32^. Thus, to test whether Notch signaling is specifically dysregulated in the spinal cord, we examined the NICD expression levels in other tissues of SMNΔ7 mice, including the whole brain and the gastrocnemius. NICD expression was unchanged in both the whole brain and gastrocnemius of SMNΔ7 mice (Supplemental Fig. 5; 9C–F), suggesting that Notch signaling was mainly dysregulated in the spinal cord. Therefore, Notch signaling may be a key factor in determining the cell type specificity in the pathological mechanism of SMA. Thus, to test whether SMN-deficient astrocytes may induce motor neuron death, we investigated whether conditioned medium from SMA-iPSC MN cultures (SMA-CM), in which the number of GFAP and S100-positive astrocytes had increased (Fig. 4A–C), had the potential to induce motor neuron death. To this end, we evaluated motor neuron cell death in the neuroblastoma spinal cord 34 (NSC-34), by combination staining with Hoechst 33342 and propidium iodide (PI; a marker of dead cells) (Fig. 4D). We found that SMA-CM increased the number of PI-positive motor neurons and the level of interleukin 1β (IL-1β), one of the key neurotoxic molecules released from astrocytes previously reported (Fig. 4E–G)^33^. Next, we performed co-immunostaining of GFAP/IL-1β using the SMA-iPSC MN culture at day 56 when we collected the WT/SMA-CM. These data indicated that IL-1β was co-localized with GFAP, suggesting that IL-1β was co-localized mainly in GFAP-positive astrocytes (Fig. 4H). Finally, to support the data in a more physiological condition, we investigated whether the astrocytes in SMNΔ7 mice express IL-1β. In this experiment, we focused on the astrocytes and oligodendrocytes, because iPSC-derived culture includes the various cell types from neural stem cells (neuron, astrocytes, oligodendrocytes). The data indicated that IL-1β was co-localized with not myelin basic protein (MBP) but with GFAP, suggesting that the astrocytes in physiological conditions also may be a key player of disease progression through expressing IL-1β, although further analysis will be needed (Fig. 4I).

### Pharmacological Inhibition of Notch Signaling Ameliorates the Astrocytic Abnormality in an SMA-Patient-Derived iPSC Culture System

**Figure 5:**
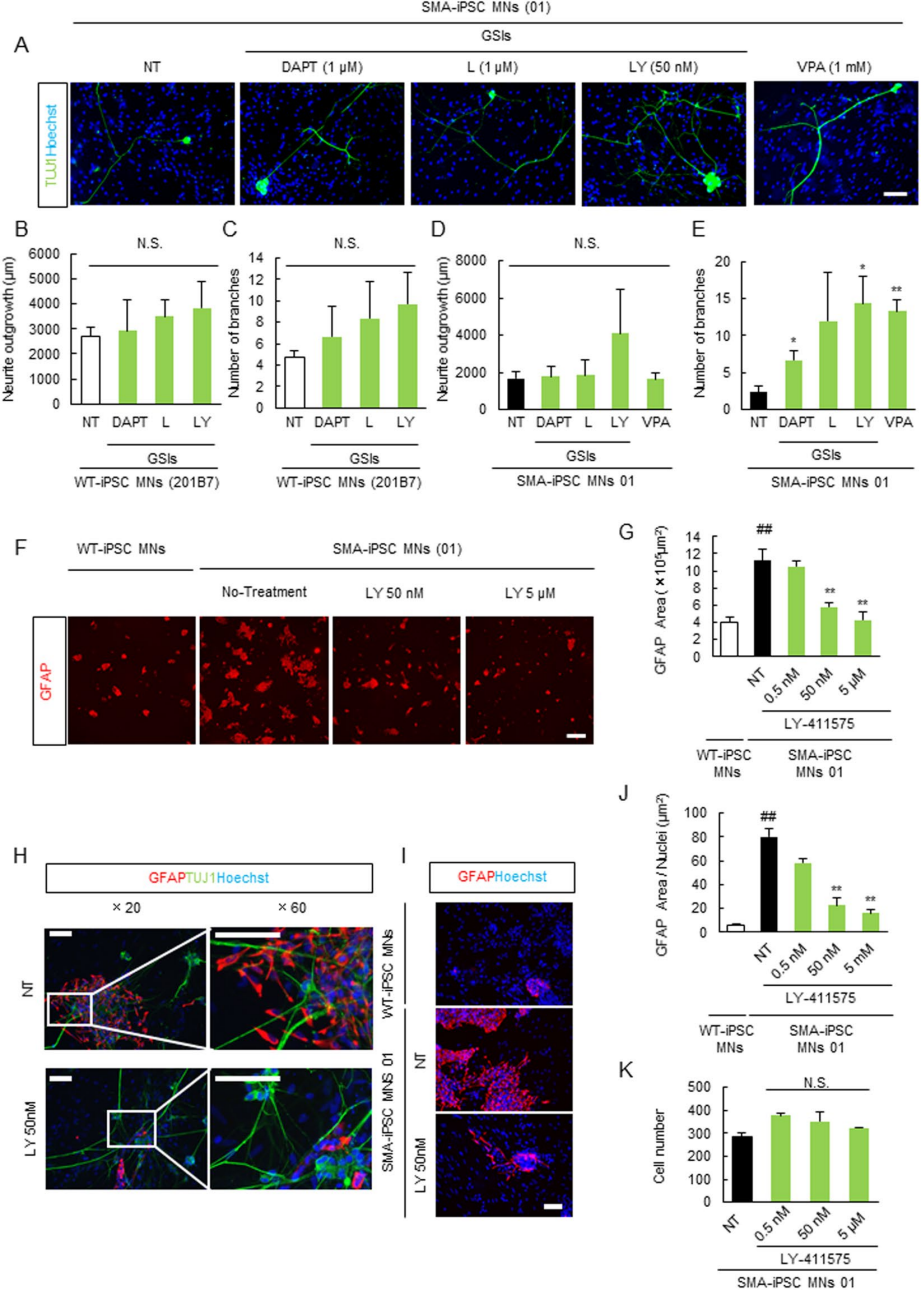
Notch inhibitor, LY-411575 inhibited the GFAP-positive astrocyte abnormality. (**A**) The assay of TUJ1-positive neurite outgrowth and branching point to identify the most potent γ-secretase inhibitor which canceled the activation of Notch signaling using SMA-iPSC MNs. Scale bar shows 100 μm. GSIs mean γ-secretase inhibitors including DAPT, L-685,458, LY-411575. (**B**,**C**) The quantitative data of TUJ1-positive neurite outgrowth (**B**) and branching point (**C**) in WT-iPSC MNs. Values represent the mean ± SEM (n = 3, Student’s *t*-test). (**D,E**) The quantitative data of TUJ1-positive neurite outgrowth (**D**) and branching point (**E**) in SMA-iPSC MNs. Values represent the mean ± SEM (n = 3, Student’s *t*-test). **p < 0.01 and *p < 0.05 versus NT (Student’s *t*-test). (**F**) Representative images of GFAP-positive astrocytes with low magnification (x2). Scale bar shows 600 μm. (**G**) The quantitative analysis of GFAP-positive area with or WT- or SMA-iPSC MNs (NT and treated with LY-411575 groups). Data represents mean ± SEM. (n = 3). ^##^*p* < 0.01 versus WT-iPSC-MNs (Student’s *t*-test) **p < 0.01 versus NT (Dunnet’s test). (**H**,**I**) Representative images of GFAP-positive astrocytes and TUJ1-positive neurite with high magnification (x 20 and x 60). Scale bar shows 50 μm. (**J**) The quantitative analysis of GFAP-positive area/nuclei with or WT- or SMA-iPSC-MNs (NT and treated with LY-411575 groups). Data represents mean ± SEM. (n = 3). ^##^*p* < 0.01 versus WT-iPSC MNs (Student’s *t*-test) **p < 0.01 versus NT (Dunnet’s test). (**K**) The quantitative analysis of cell number using LY-411575, which inhibits Notch signaling. The cells were treated with LY-411575 (0.5 nM, 50 nM and 5 µM). LY-411575 had no change of Hoechst 33342-positive cell number in SMA-iPSC MNs. Values represent the mean ± SEM. (n = 3 or 4).

In a previous report, inhibition of Notch signaling enhanced the differentiation of human embryonic stem cells (hESCs) into motor neurons, and N-[N-(3, 5-difluorophenacetyl)-l-alanyl]-S-phenylglycine t-butyl ester (DAPT), a Notch signaling inhibitor, increased β-III tubulin (TUJ1)-positive neuron immunostaining in an hESC culture^34^. Therefore, Notch inhibition may be a key component in motor neuron and astrocyte differentiation in human stem cell culture systems. To test this hypothesis, we first identified the most potent Notch inhibitor by TUJ1 immunocytochemistry of WT or SMA-iPSC MNs treated with DAPT, L-685,458, or LY-411575. LY-411575 induced the highest increases in axon length and number of branching points in TUJ1-positive cells specifically in SMA01-iPSC MNs (Fig. 5A–E). Therefore, we used LY-411575 as the Notch inhibitor in subsequent experiments. To elucidate the involvement of Notch signaling in the GFAP-positive astrocyte abnormality in our SMA cell culture system, we investigated the effect of LY-411575 on the increase in GFAP immunoreactivity. LY-411575 decreased the GFAP-positive area in a concentration-dependent manner (Fig. 5F–H). Further, the GFAP-positive area per nucleus was also decreased and the cell number was not altered by LY-411575 (Fig. 5I–K), ruling out the possibility that LY-411575 induced astrocyte death.

### Pharmacological Inhibition of Notch Signaling Partially Rescues Motor Function Deficits in SMNΔ7 Mice

**Figure 6:**
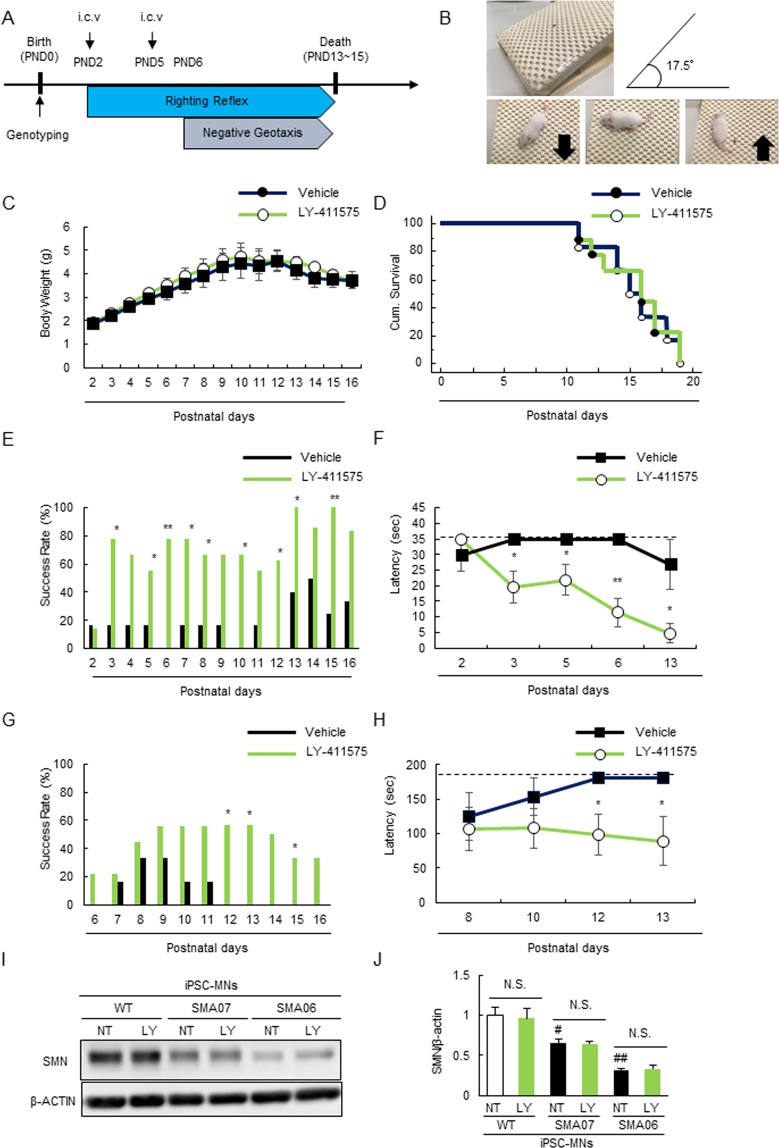
LY-411575 ameliorates the motor functional deficits of SMNΔ7 mice. (**A**) The scheme for the LY-411575 administration. Injection of either vehicle or LY-411575 (1.4 ng/body) were administrated at PND2 and PND5. Righting reflex and negative geotaxis assay was performed from PND2 and PND6, respectively. (**B**) The representative image of negative geotaxis test. (**C**) The body mass curve of SMNΔ7 mice during treatment with LY-411575 did not differ from those of vehicle-treated mice. Data represents mean ± SEM (n = 6 or 9). (**D**) The effects of LY-411575 (1.4 ng/body, i.c.v.) on survival in SMNΔ7 mice. The mean survival of vehicle-treated group and LY-411575-treated group mice were 15.56 ± 0.97 (n = 6) and 15.50 ± 1.18 (n = 9), respectively. (**E**) The success rate SMNΔ7 mice in acquiring righting reflex tested. **p < 0.01 and *p < 0.05 versus vehicle-treated group (one-tailed chi-square test). (**F**) The latency of righting reflex tested using SMNΔ7 mice at PND2 (pre administration), 3, 5, 6 (post administration) and 13 (late stage). Data represents mean ± SEM (n = 6 or 9). **p < 0.01 and *p < 0.05 versus vehicle-treated group (Student’s *t*-test). (**G**) The success rate SMNΔ7 mice in acquiring negative geotaxis tested. **p < 0.01 and *p < 0.05 versus vehicle-treated group (one-tailed chi-square test). (**H**) The latency of negative geotaxis tested using SMNΔ7 mice at PND8, 10 12 and 13 (late stage). Data represents mean ± SEM (n = 6 or 9). *p < 0.05 versus vehicle-treated group (Student’s *t*-test). (**I**,**J**) SMN expression in WT/SMA06 and 07-iPSC-MNs. Values represent the mean ± SEM. (n = 3). ^##^*p* < 0.01 ^#^ and *p* < 0.05 versus WT-iPSC-MNs (Student’s *t*-test). The cropped blots are used in this Figure and full-length blots are presented in Supplemental Figure 6.

To evaluate the impact of LY-411575 on disease phenotype, we examined whether LY-411575 could rescue the motor function deficits in SMNΔ7 mice. We injected mice with LY-411575 intracerebroventricularly (i.c.v.) at PND2 and 5, and assessed their weights, motor functions, and longevity daily (Fig. 6A,B). SMNΔ7 mice treated with LY-411575 showed improved motor function (as assessed by the righting reflex, Fig. 6E,F; and negative geotaxis test, Fig. 6G,H), although their weights and survival lengths were unaffected (Fig. 6C,D). In addition, the limited effects of LY-411575 on the survival of SMNΔ7 mice may be explained by its inability to elevate SMN protein levels (Fig. 6I,J).

### Pharmacological Inhibition of Notch Signaling Inhibits Abnormal Astrocyte Numbers in SMNΔ7 Mice

**Figure 7:**
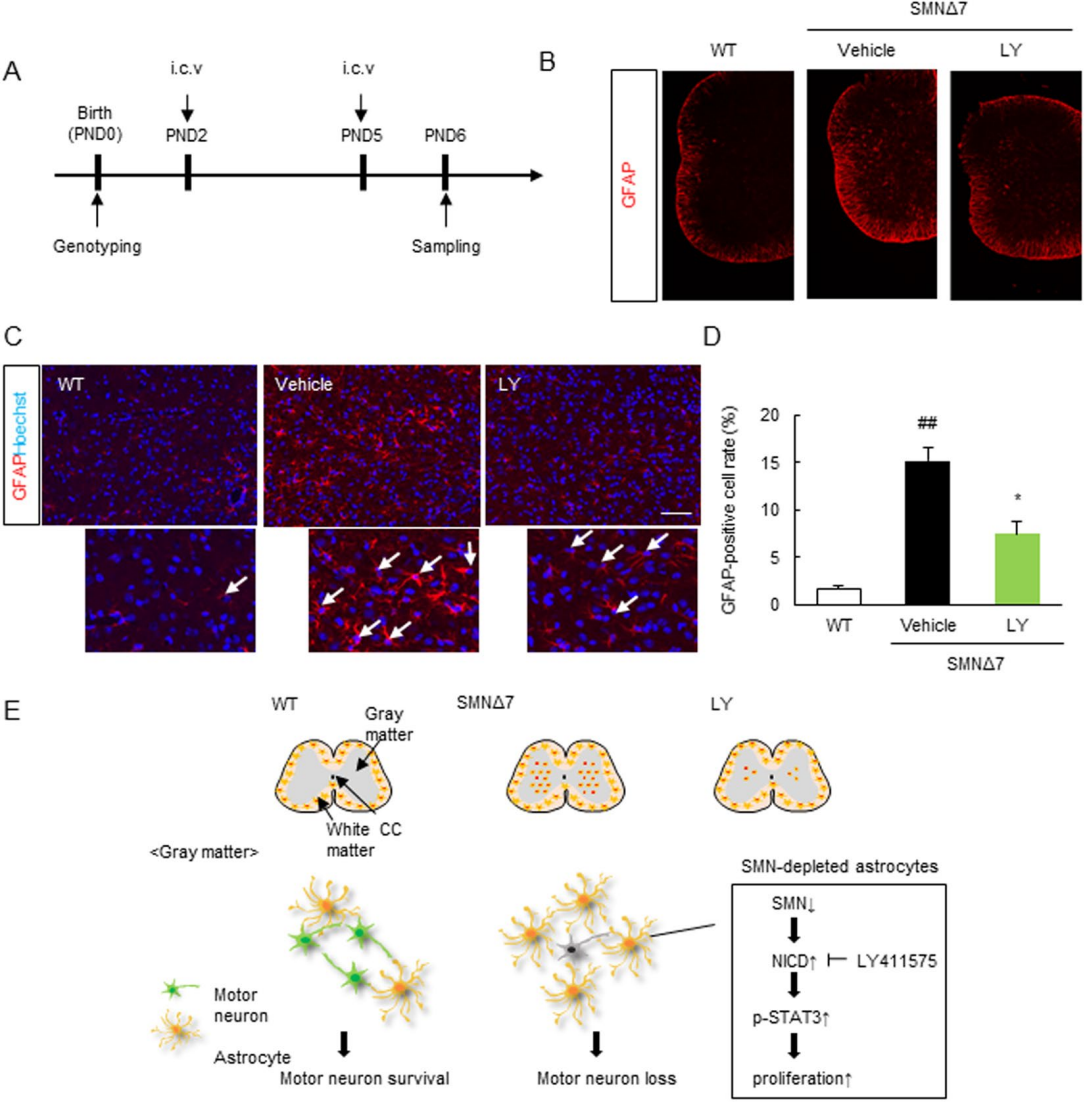
LY-411575 inhibits the GFAP-positive astrocytes in spinal cord of SMNΔ7 mice. (**A**) The scheme for the GFAP-positive cell assay when Notch signaling was inhibited by LY-411575. Injection of either vehicle or LY-411575 (1.4 ng/body) were administrated at PND2 and PND5 and sampling was performed at PND6. (**B**) Representative fluorescence images (x 4) of GFAP in the whole spinal cord of WT mice, SMNΔ7 mice and SMNΔ7 mice treated by LY-411575. Scale bar shows 50 μm. (**C**) Representative images of GFAP-positive astrocytes in the gray matter of spinal cord with high magnification (x 20). Scale bar shows 100 μm. (**D**) The quantitative analysis of GFAP-positive cells/nuclei with or WT mice, SMNΔ7 mice and SMNΔ7 mice treated by LY-411575. Data represents mean ± SEM. (n = 3). ^##^*p* < 0.01 versus WT mice and *p < 0.05 versus vehicle (Student’s *t*-test). (**E**) The putative mechanism underlying increase in astrocyte number in gray matter of SMA-spinal cord. SMN-depletion induced the dysregulation of Notch signaling, which promoted astrocyte proliferation in spinal cord of SMNΔ7 mice. Notch inhibition by LY-411575 inhibited the astrocytic abnormality.

Finally, to investigate the effect of pharmacological inhibition of Notch signaling on the SMA-related astrocytic abnormality *in vivo*, we counted GFAP-positive astrocytes in the spinal cord GM of WT and SMNΔ7 mice. SMNΔ7 mice were treated with LY-411575 at PND2 and 5, followed by immunohistochemistry of the lumbar spinal cord at PND6 (Fig. 7A). LY-411575 (1.4 ng/body/day) significantly (*p* = 0.020, Student *t*-test) reduced the number of GFAP-positive cells in SMNΔ7 mice compared to that in untreated SMNΔ7 mice (Fig. 7B–D). These results indicated that the SMA-related astrocyte abnormality was likely mediated by dysregulated Notch signaling (Fig. 7E).

## Discussion

Some evidence indicated that astrogliosis is defined as the morphological change in response to injury, while astrogenesis is defined as the proliferation independent of a response to injury^35–37^. It is unclear whether astrogliosis defined as increased GFAP immunoreactivity reflects a faster GFAP production rate, an increase in the number or average size of astrocytes, or a combination thereof^38^. In addition, regardless of which of the above phenomena underlie the elevated GFAP immunoreactivity in SMA model systems, its causative mechanisms remain unclear, as is whether the astrocytic aberration might be targeted in the development of SMA therapeutics. The results of the present study suggest that chronic SMN depletion during developmental stages might cause an abnormal increase in astrocytes in early-onset SMA via Notch signaling dysregulation. In addition, Notch signaling was critically involved in the SMA pathology in a mouse model, indicating that manipulation of the Notch pathway may be a promising approach to treat patients with SMA.

We found that the number of GFAP-positive astrocytes (i.e., terminally differentiated and fibrous astrocytes) was increased in the spinal cord GM, which contains the cell bodies of numerous motor neurons, but not the WM of SMNΔ7 mice (Fig. 1A–D). Tien *et al*. described a pattern of astrocyte migration, proliferation, and differentiation under physiological conditions. In this report, astrocyte precursor cells extensively migrate from the ventricular zone (VZ) around the central canal (CC) to the mantle zone, where they differentiate, during embryonic day 15-PND3^39^. It is possible that SMN-deficient astrocytes proliferate on their own because the number of S100-positive cells (protoplasmic astrocytes) were increased only in the CCs of SMNΔ7 mice (Fig. 1E–I) and GFAP-Ki67-positive cells were increased (Fig. 2N,O), suggesting that this phenomena of remaining of proliferative astrocytes in SMA pathology may indicate astrogenesis. Further study is needed to determine the features of SMN-deficient astrocytes underlying the astrocytic pathology in SMA.

We also showed that the NICD-STAT3-GFAP/S100 axis, which promotes astrocyte differentiation and proliferation, was upregulated in the spinal cords of SMNΔ7 mice and in an SMA-patient-derived stem cell model (Fig. 2; Fig. 3). Furthermore, pharmacological inhibition of the Notch signal using LY-411575 may ameliorate motor functional deficits by abrogating the increase in GFAP-positive astrocytes, albeit without affecting the survival time (Fig. 5F–K; Fig. 6C–H; Fig. 7B–D). Taken together, our results suggest that Notch signaling is involved in SMA pathology and might be used as a therapeutic target of SMA treatments.

Recently, the first successful clinical trial of an antisense oligonucleotide (ASO; nusinersen, commercial name Spinraza^®^) therapy for SMA was completed by Ionis Pharmaceuticals and Biogen^40,41^. The findings revealed that nusinersen increases full-length *SMN2* transcription^42^. Based on the robust clinical data, nusinersen was approved for all types of SMA, although the motor function of about half of the patients with SMA was not improved. Therefore, we propose the other therapies that inhibit astrocyte abnormalities and motor neuron degeneration independent of SMN expression in this study. Based on our findings, Notch signaling inhibition may be a promising target for combination therapy with nusinersen for SMA.

## Material and Methods

### Ethics Statement

The generation and pathological analysis (including human gene analysis) of patient-derived iPSC in this study were approved by the Ethical Review Committee of the National Hospital Organization, Nagara Medical Center (approved No. 26-15), and Gifu Pharmaceutical University (approved No. 27-213). During this study, established human stem cells were handled according to the Revisions of the Guidelines for Clinical Research Using Human Stem Cells by the Ministry of Health, Labor, and Welfare of Japan. Written informed consents were obtained from all SMA subjects for publication of this report. While the 201B7 line was provided Dr. K. Osafune (Kyoto University).

### Animals

A pair of heterozygous SMNΔ7 mice (*mSmn*^+/−^, *SMN2*^+/+^, *SMNΔ*7^+/+^) were purchased from Jackson Laboratory (Bar Harbor, ME, USA). The homozygous SMNΔ7 mice (affected and referred to as “SMNΔ7 mice”; *mSmn*^−/−^, *SMN2*^+/+^, *SMNΔ*7^+/+^) was generated by cross-bleeding of heterozygous SMNΔ7 mice. The SMNΔ7 mice indicated the short life span and motor function deficits previously reported^43,44^. All mice were housed at 24 °C under a 12 h light–dark cycle (lights on from 8:00 to 20:00) and had *ad libitum* access to food and water. All experiments were approved and monitored by the Institutional Animal Care and Use Committee of Gifu Pharmaceutical University, and were performed after approval by the Bioethics and Biosafety Committee of Gifu Pharmaceutical University (approval No. 2014-236, 2015-154, 2015-155, 2016-163, 2017-090, 2017-196). All procedures relating to animal care and treatment confirmed to animal care guidelines issued by the National Institutes of Health. Offspring were genotyped using PCR assays of tail DNA to identify WT (unaffected mice; *mSmn*^+/+^*SMN2*^+/+^*SMN*Δ7^+/+^), heterozygous SMNΔ7, and SMNΔ7 mice. The target gene was amplified by 35 cycles of PCR using the following 3 primers: 5′-CTCCGGGATATTGGGATTG -3′, 5′-GGTAACGCCAGGGTTTTCC-3′; 5′-TTTCTTCTGGCTGTGCCTTT-3′.

### LY-411575 Administration

LY-411575 (Xcess Biosciencess, San Diego, CA, USA) was diluted in dimethyl sulfoxide (DMSO; Nacalai Tesque, Kyoto, Japan) and administered (1.4 ng/body) by i.c.v. injection using a 34-gauge needle (TERUMO, Tokyo, Japan) as described previously^45^. WT mice received equal volumes of vehicle alone.

### Behavioral Analysis

For survival studies, body weight measurements and behavioral tests were performed daily. Mice with 30% weight loss and unable to right were euthanized with carbon dioxide. Righting time was defined as the time it took a pup to turn over to its front after being placed completely on its back (maximum 35 s). In the negative geotaxis test, each pup was placed on a 17.5° incline with its head pointing down. The latency for turning 180° was recorded (maximum 180 s). Righting times and negative geotaxis responses were measured starting from PND2 and 6. These behavioral analyses were based on previous reports^43,46^.

### Spinal Motor Neuron iPSC Culture

In this study, we adopted a previously reported modified protocol that mimics normal motor neuron generation^11,47^.

### Immunostaining

Immunohistochemistry (IHC) was performed as follows. Mice anesthetized with sodium pentobarbital (5 mg/kg intraperitoneally) were perfused with saline for 3 min and then with 4% paraformaldehyde (Nacalai Tesque, Kyoto, Japan) for 6 min. Lumbar spinal cord segments (L1-5) were dissected and post-fixed in the same fixative for 24 h at 4 °C. The tissues were equilibrated in a 25% sucrose solution for 24 h and quickly embedded in optimal cutting temperature compound (Sakura Finetechnical Co., Ltd., Tokyo, Japan). Finally, 10 μm transverse sections were cut on a cryostat and placed on glass slides (MAS COAT; Matsunami Glass Ind., Ltd., Osaka, Japan). The spinal cord sections were blocked with a non-immune serum mix (goat and horse sera [Vector Labs, Burlingame, CA, USA]) for 1 h and incubated with primary antibodies at 4 °C overnight. For mouse antibodies, the M.O.M immunodetection kit (Vector Labs) was used for blocking and incubation solution preparation. After primary antibody (listed in Supplemental Fig. 10) binding, the sections were incubated with a secondary antibody and Hoechst 33342 (1:1,000 dilution; Invitrogen, Carlsbad, CA, USA) for 1 h. Finally, the sections were mounted in Fluoromount (Diagnostic BioSystems, Pleasanton, CA, USA). The secondary antibodies were Alexa Fluor^®^546 goat anti-mouse IgG or Alexa Fluor^®^488 goat anti-rabbit IgG (1:1,000 dilution; both Invitrogen). Images of these sections were obtained using a BZ-9000 HS all-in-one fluorescence microscope (Keyence, Osaka, Japan). Immunocytochemistry (ICC) was peformed using previous protocols^11^. Primary antibody is listed in Supplemental Fig. 10.

### Western Blot Analysis

Samples were lysed using radioimmunoprecipitation assay buffer containing 50 mM Tris hydrochloride, 150 mM sodium chloride, 0.5% sodium deoxycholate, 0.1% sodium dodecyl sulfate (SDS), 1% Igepal CA-630, and protease and phosphatase inhibitor cocktails (Sigma-Aldrich, St. Louis, MO, USA). The lysates were centrifuged at 12,000 × *g* for 10 min, and the supernatants were analyzed. Protein concentrations were determined using the bicinchoninic acid protein assay kit (Pierce Biotechnology, Rockford, IL, USA) with bovine serum albumin as the standard. Equal volumes of lysate and sample buffer containing 20% 2-mercaptoethanol (Wako, Osaka, Japan) were mixed, and the proteins were separated using 5–20% SDS-polyacrylamide gel electrophoresis (Wako). The proteins were transferred to the Immun-Blot polyvinylidene fluoride membrane (Bio-Rad Laboratories, Hercules, California, USA). The primary antibodies used are listed in Supplemental Fig. 10; the secondary antibodies were mouse anti-goat, goat anti-rabbit and goat anti-mouse IgGs conjugated to horseradish peroxidase (Thermo Fisher Scientific, Waltham, MA, USA, dilution for all secondary antibodies 1:2,000). The immunoreactive bands were visualized using a chemiluminescent substrate (ImmunoStar^®^ LD; Wako Pure Chemicals). The band intensities were measured using the LAS-4000 image analyzer (Fuji Film, Tokyo, Japan).

### Statistical Analysis

Data are presented as means ± standard error of the mean. Statistical significance was evaluated using the two-tailed Student *t*-test or two-way analyses of variance (ANOVA) followed by the two-tailed Student *t*-test, using Statistical Package for the Social Sciences 16.0 J (SPSS Japan, Inc., Tokyo, Japan). A chi-square test of independence was calculated comparing the success rate of righting reflex test and negative geotaxis test in WT and SMNΔ7 mice. For calculating the success rate, 2 × 2 contingency tables were generated and analyzed by Pearson χ^2^ with the strength of association being determined by φ. The statistical analysis of the cumulative probability of survival was performed using Kaplan-Meier life test. In Kaplan-Meier statistical analysis, the log-rank test was used for the comparison of the survival curve. Statistical analysis of survival was performed with the value of p < 0.05 being considered to indicate statistical significance.

## Supplementary information

Supplemental Figure
