# Supplementary material for: Notch Signaling Mediates Astrocyte Abnormality in Spinal Muscular Atrophy Model Systems
Source: Sci Rep. 2019 Mar 6;9:3701. doi: 10.1038/s41598-019-39788-w (PMC6403369; doi:10.1038/s41598-019-39788-w)
Supplement: Supplementary file 1 — Supplemental Figure [file 41598_2019_39788_MOESM1_ESM.pdf]

Notch Signaling Mediates Astrocyte Abnormality in Spinal Muscular Atrophy Model Systems

Kazuki Ohuchi[1, 2], Michinori Funato[2], Yuta Yoshino[1], Shiori Ando[1, 2], Satoshi Inagaki[1, 2], Arisu Sato[1, 2], Chizuru Kawase[2], Junko Seki[2], Toshio Saito[3], Hisahide Nishio[4], Shinsuke Nakamura[1], Masamitsu Shimazawa[1], Hideo Kaneko[2], Hideaki Hara[1]

[1]Molecular Pharmacology, Department of Biofunctional Evaluation, Gifu Pharmaceutical University, Gifu, Japan

[2]Department of Clinical Research, National Hospital Organization, Nagara Medical Center, Gifu, Japan

[3]Department of Neurology, Toneyama National Hospital, Osaka, Japan.

[4]Department of Occupational Therapy, Faculty of Rehabilitation, Kobe Gakuin University, Kobe, Japan.

*For reprints and all correspondence: Professor H. Hara, Ph.D., R.Ph., Molecular Pharmacology, Department of Biofunctional Evaluation, Gifu Pharmaceutical University, 1-25-4 Daigaku-nishi, Gifu 501-1196, Japan.

Tel & Fax: +81-58-230-8126

e-mail: hidehara@gifu-pu.ac.jp

Supplemental Materials and Methods

Karyotyping

Karyotyping of SMA-iPSC by chromosomal G-band analysis was carried out by Nihon Gene Research Laboratories, Japan.

Alkaline phosphatase (ALP) staining

ALP staining was performed with an Alkaline Phosphatase Detection Kit (Chemicon, Temecula, CA, USA) according to previous report [10].

1 Ohuchi K, Funato M, Kato Z, et al. Established Stem Cell Model of Spinal Muscular Atrophy Is Applicable in the Evaluation of the Efficacy of Thyrotropin-Releasing Hormone Analog. Stem cells translational medicine. 2016 Feb;5(2):152-63.

Supplemental Figure 1

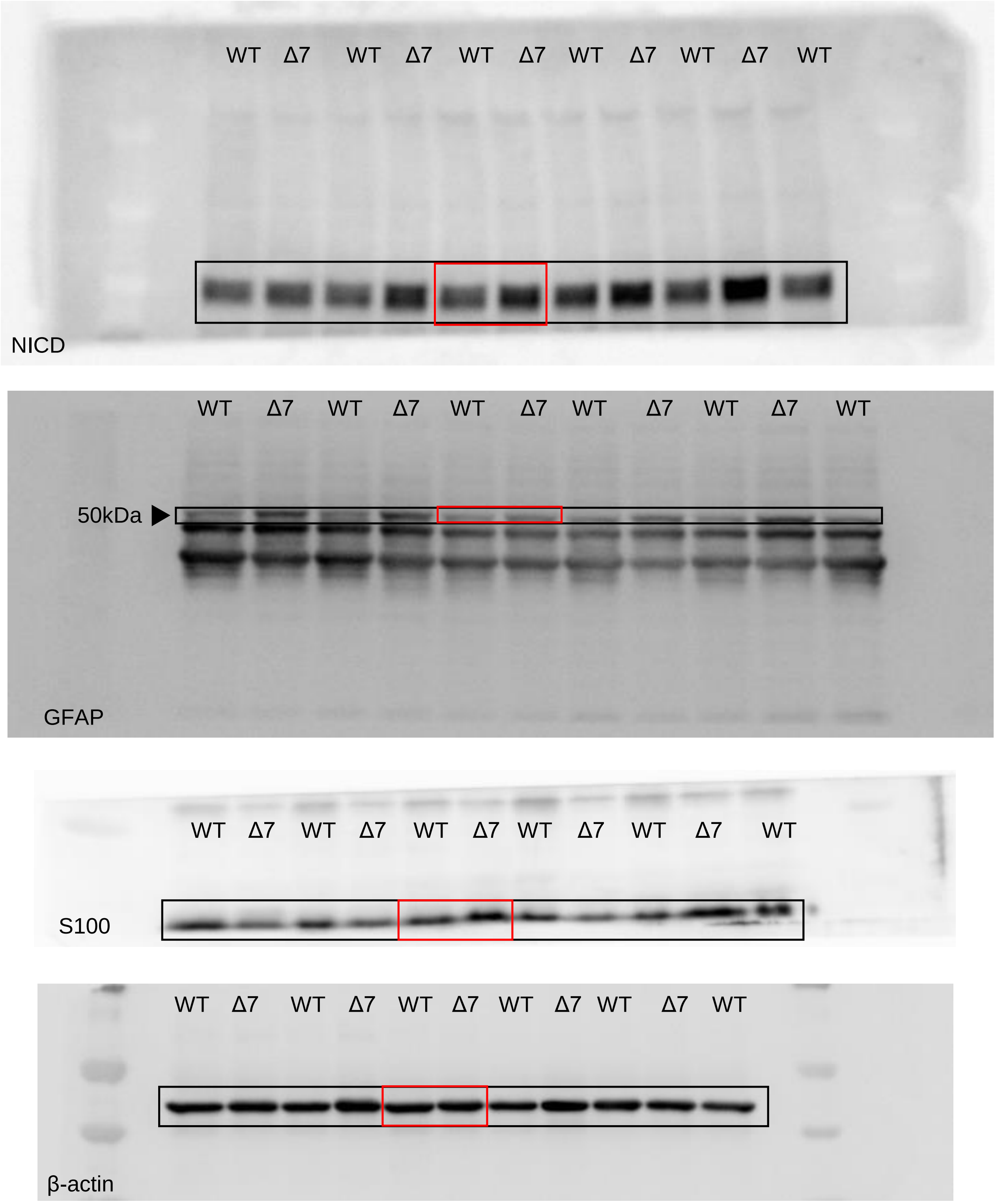

Supplemental Figure 1. Western blot analysis of NICD, GFAP, S100 expression in the spinal cords of WT and SMNΔ7 mice at PND11.

Representative immunoblot showing the NICD, GFAP, S100 expression level in the spinal cords of WT and SMNΔ7 mice. Red squares in the full-length blots are used for the cropped blots in Figure 2A. Black squares in the full-length blots are used for the quantitative analysis in Figure 2B-D.

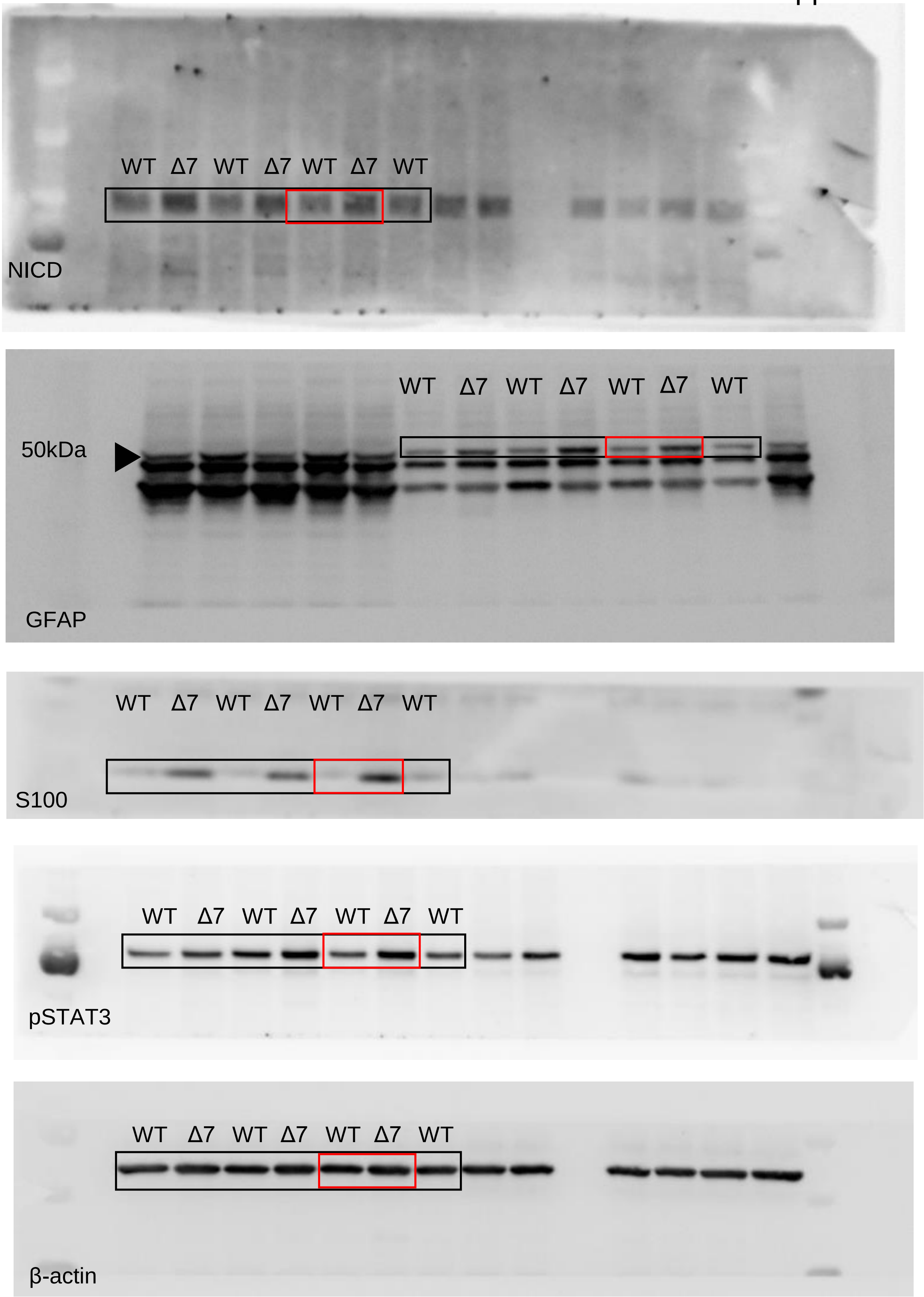

Supplemental Figure 2. Western blot analysis of NICD, GFAP, S100 expression in the spinal cords of WT and SMNΔ7 mice at PND5.

Representative immunoblot showing the NICD, GFAP, S100 expression level in the spinal cords of WT and SMNΔ7 mice. Red squares in the full-length blots are used for the cropped blots in Figure 2E. Black squares in the full-length blots are used for the quantitative analysis in Figure 2F-I.

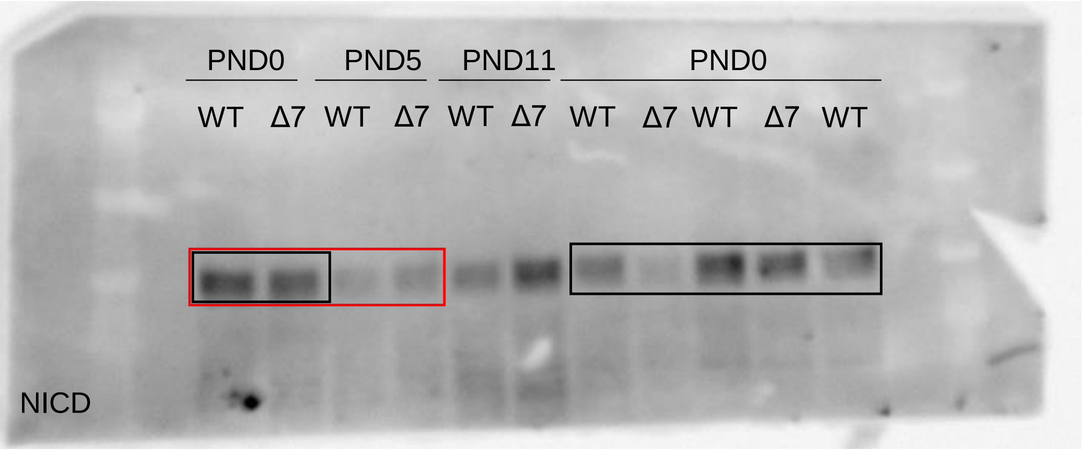

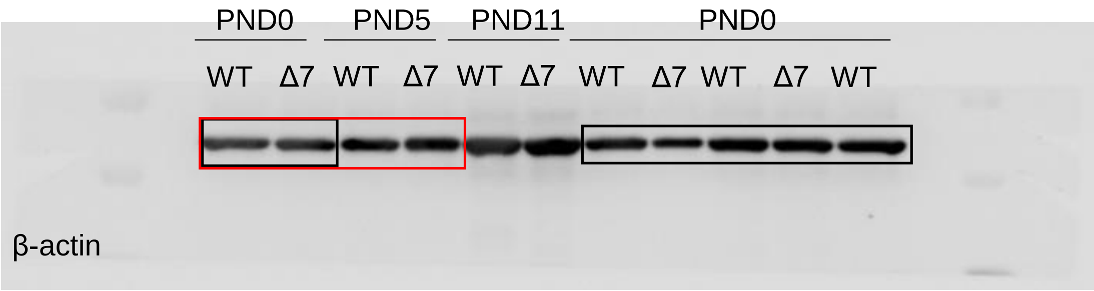

Supplemental Figure 3. Western blot analysis of NICD expression in the spinal cords of WT and SMNΔ7 mice at PND0.

Representative immunoblot showing the NICD expression level in the spinal cords of WT and SMNΔ7 mice at PND0. Red squares in the full-length blots are used for the cropped blots in Figure 2J. Black squares in the full-length blots are used for the quantitative analysis in Figure 2K.

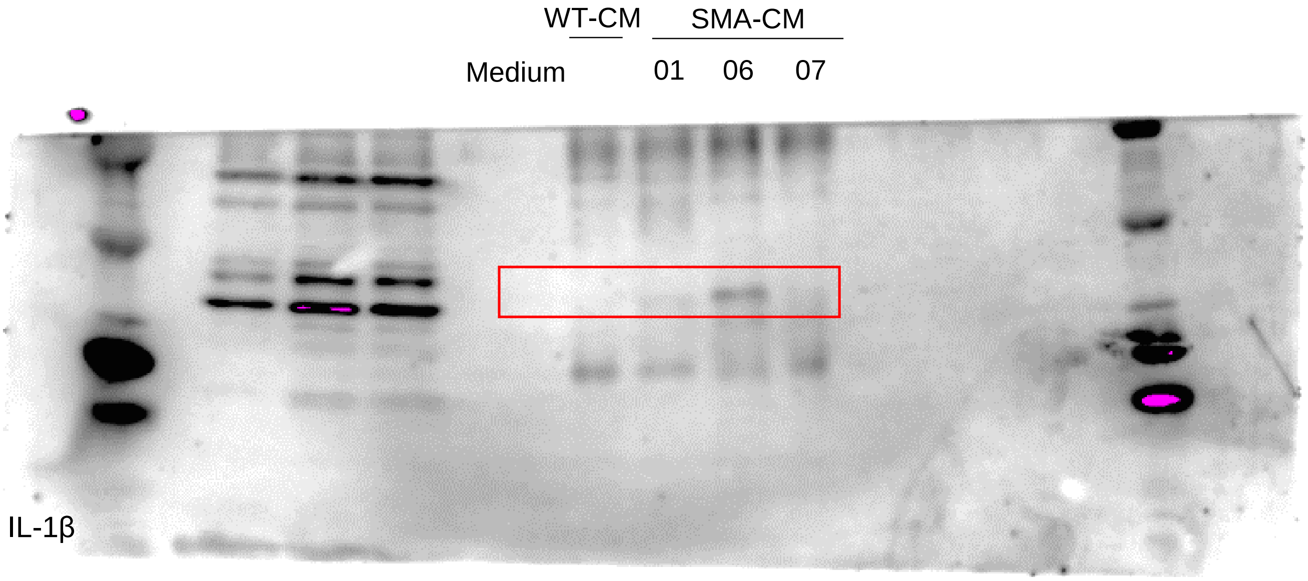

Supplemental Figure 4. Western blot analysis of IL-1β expression in conditioned medium from WT and SMA-iPSC cultures (WT/SMA-CM).

Representative immunoblot showing the IL-1β expression level in the WT and SMA-CM. Red squares in the full-length blots are used for the cropped blots in Figure 4G.

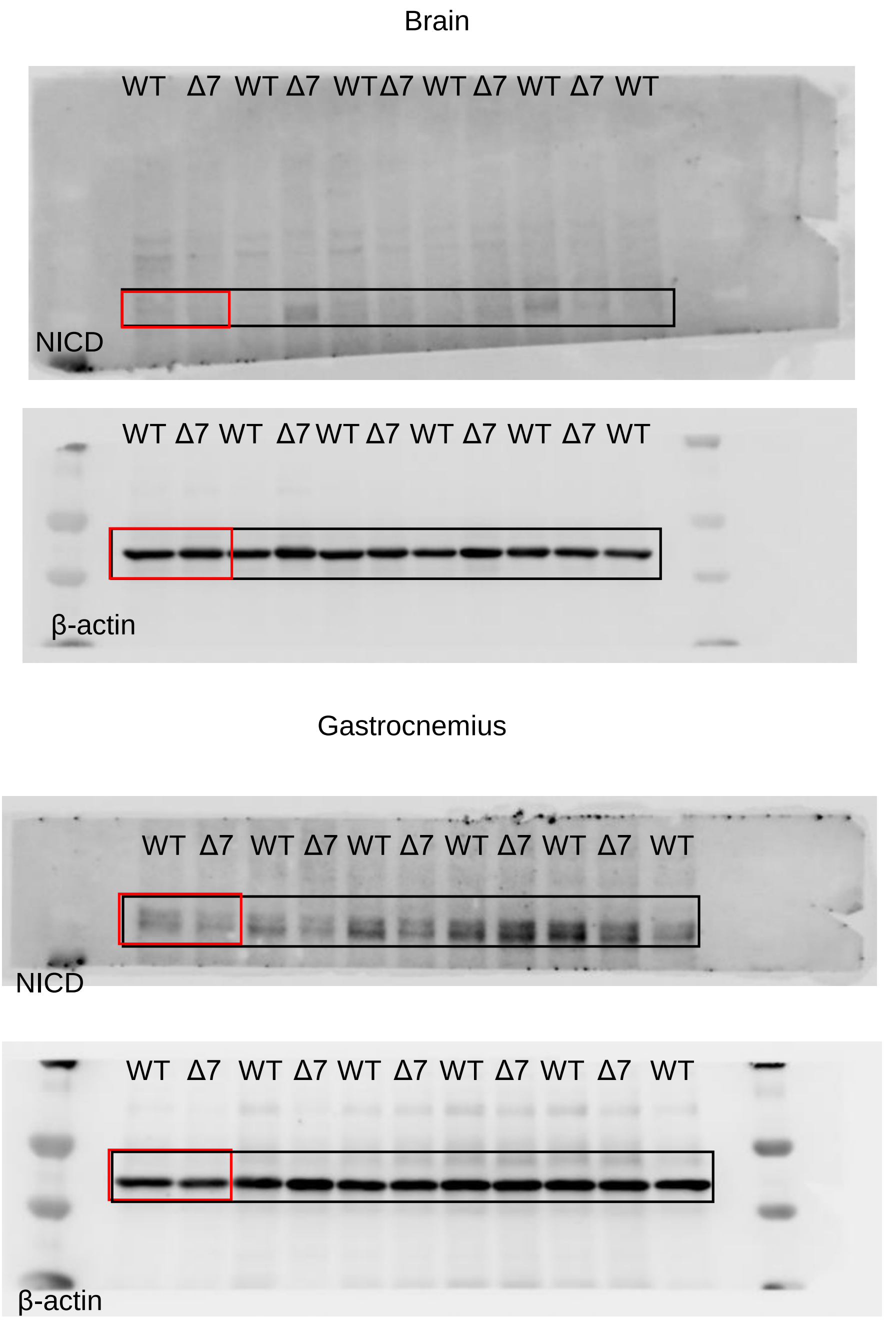

Supplemental Figure 5. Western blot analysis of NICD expression in the Brain and Gastrocenemius of WT and SMNΔ7 mice at PND11.

Representative immunoblot showing the NICD expression level in the Brain and Gastrocenemius of WT and SMNΔ7 mice. Red squares in the full-length blots are used for the cropped blots in Supplemental Figure 9C-D. Black squares in the full-length blots are used for the quantitative analysis in Supplemental Figure 9E-F.

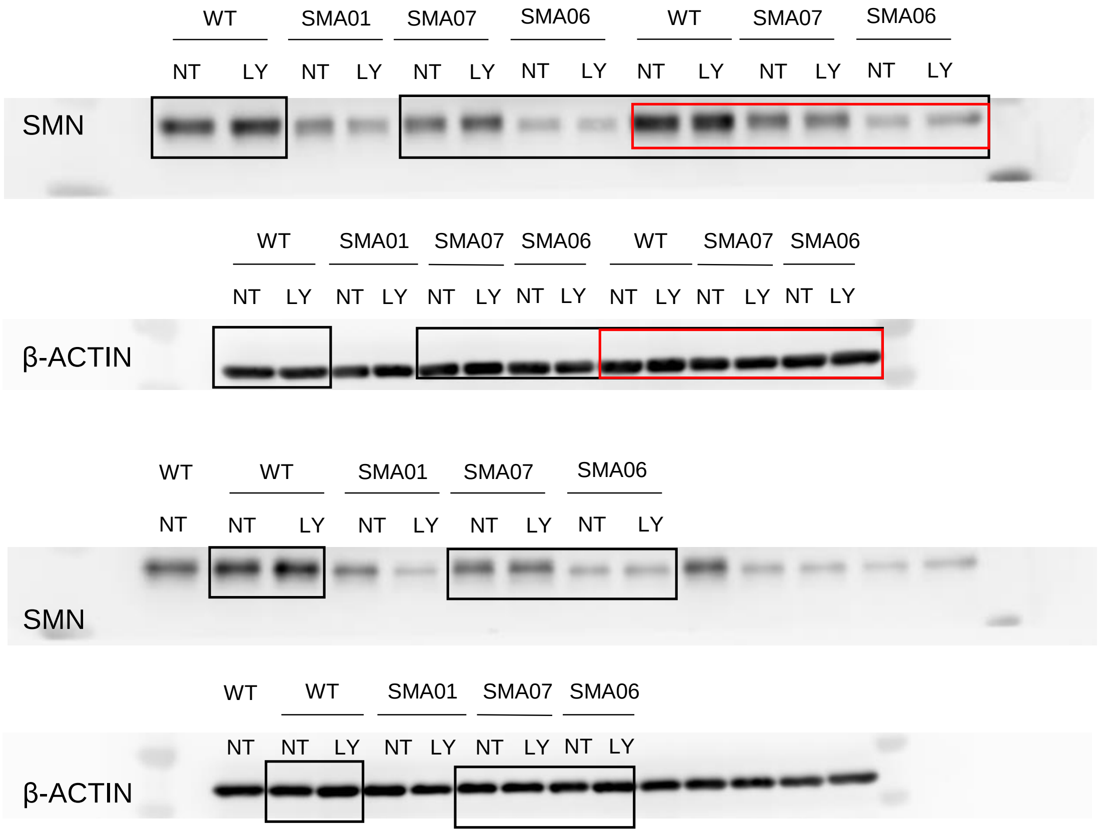

Supplemental Figure 6. Western blot analysis of SMN expression in the WT/SMA-iPSC-MNs. Representative immunoblot showing the SMN expression level WT/SMA-iPSC MNs. Red squares in the full-length blots are used for the cropped blots and black squares in the full-length blots are used for the quantitative analysis in Figure 6I-J.

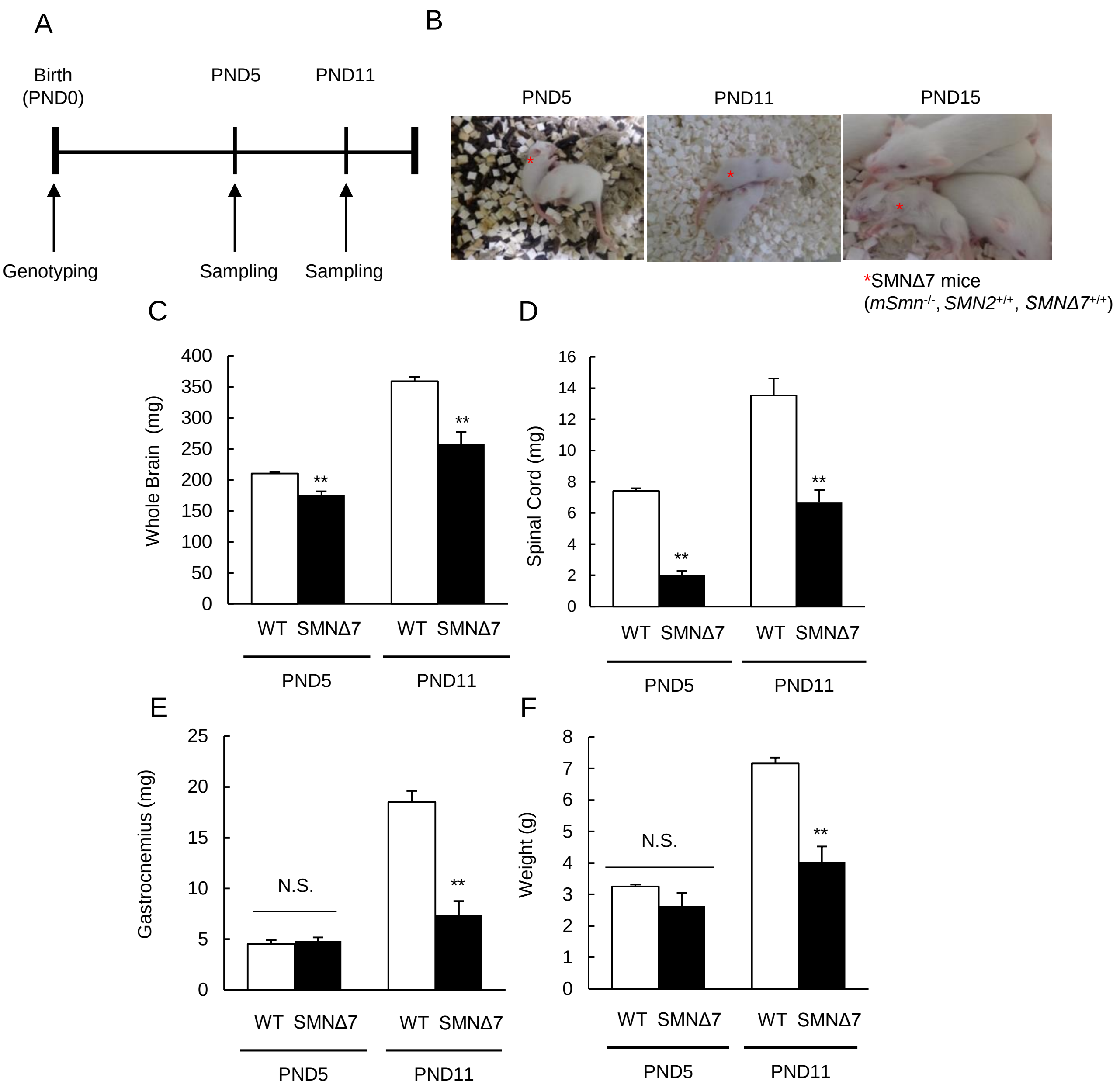

Supplemental Figure 7. Central nervous degeneration in SMNΔ7 mice precedes gastrocnemius atrophy. (A) The sampling point of brain, spinal cord and gastrocnemius in WT and SMN**Δ7** mice. (B) The typical images of WT and SMN**Δ7** mice at PND5, 11 and 15. (C-F) The quantitative analysis for the weight of brain (C), spinal cord (D), gastrocnemius (E) and body weight (F) of WT and SMN**Δ7** mice at PND5 and 11. Data represents mean ± SEM. (n = 3 or 4, PND5; n = 5 or 6, PND11). **, $p < 0.01$ versus WT mice (Student's *t*-test).

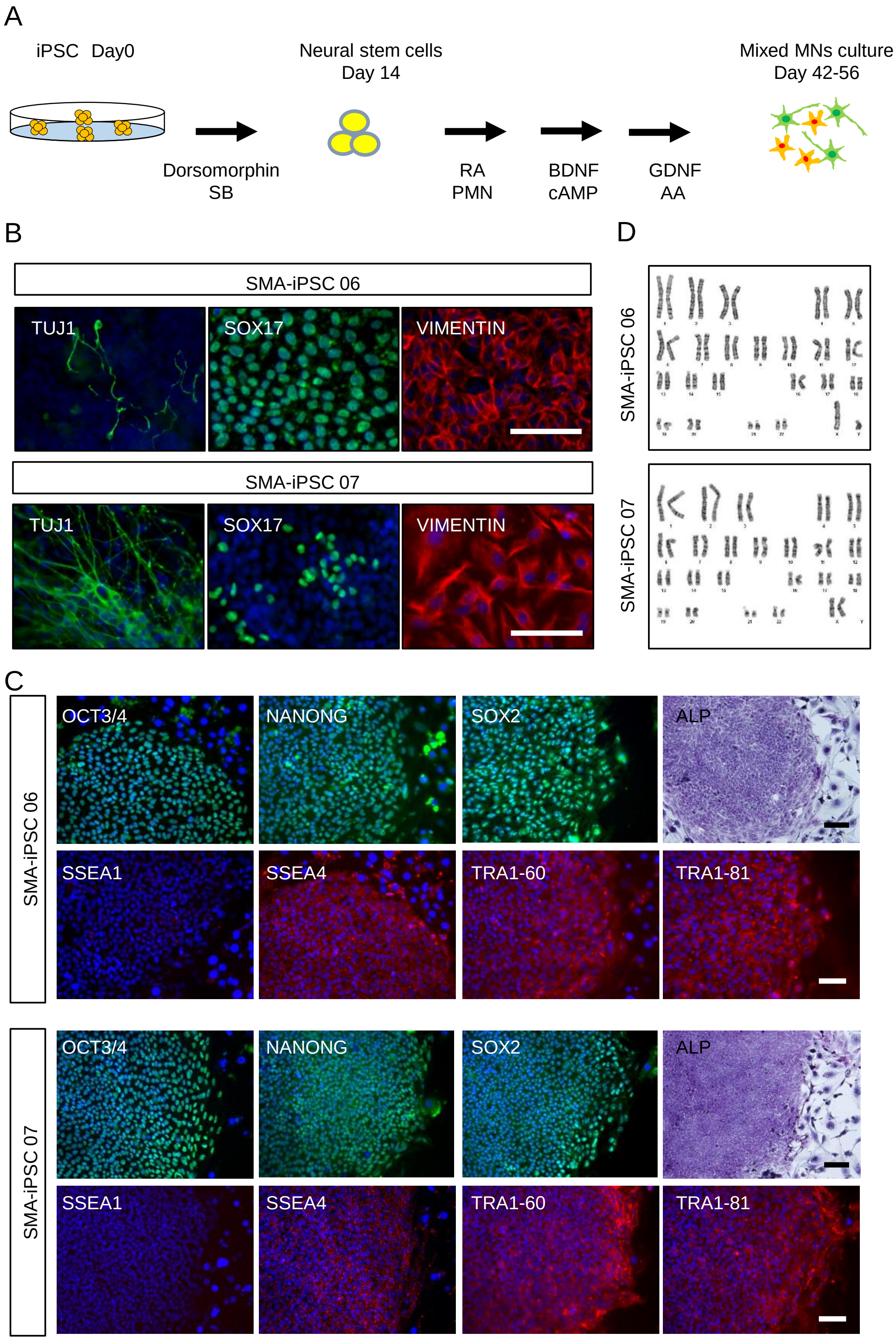

Supplemental Figure 8. Proof of iPSCs derived from SMA patients (06 and 07)

(A) The scheme of spinal motor neuron differentiation from iPSCs. (B) Expression of ectoderm (TUJ1), endoderm (SOX17) and mesoderm (VIMENTIN) markers in SMA-iPSC by embryonic body formation. Scale bars = 50 µm. (C) ALP enzymatic activities in SMA-iPSC. Scale bars shows 200 µm. Expression of pluripotent markers including OCT3/4, NANONG, SOX2, SSEA4, TRA-1-60, TRA-1-81. Scale bars shows 100 µm. (D) The karyotypic analysis. No karyotypic abnormalities were found in SMA-iPSC (SMA06 and SMA07) used in this study.

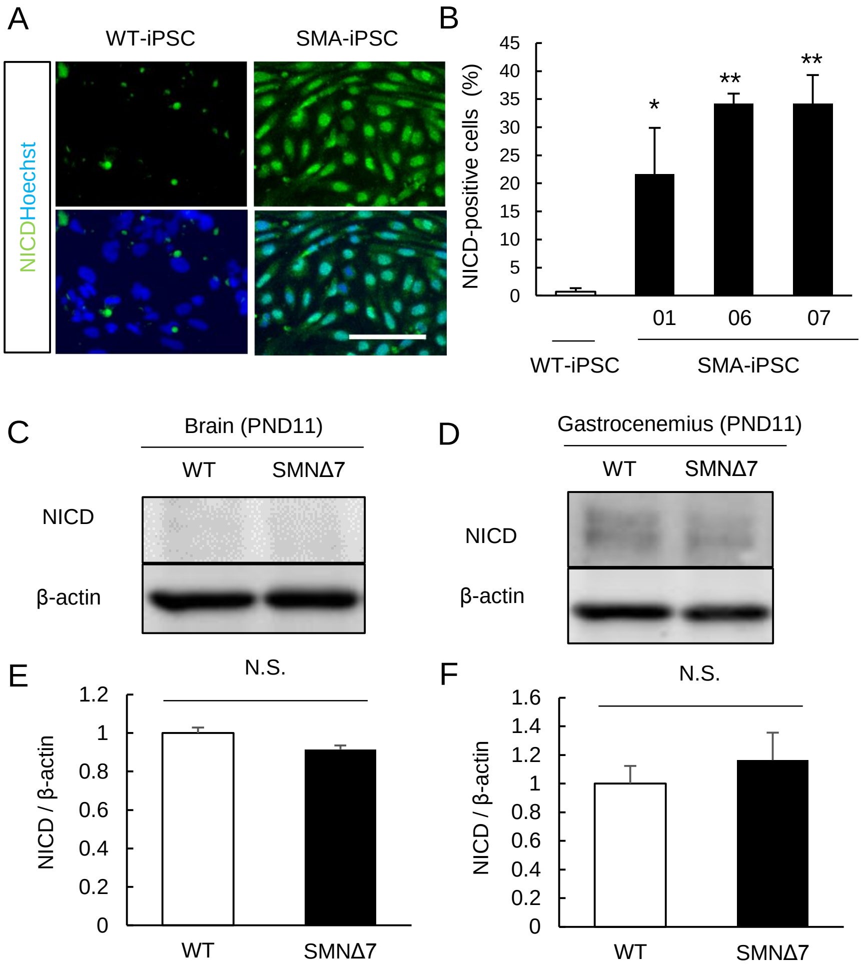

Supplemental Figure 9. Notch signaling is dysregulated specifically in spinal cord.

(A) The typical images of WT and SMA-iPSC culture at 17 days. Scale bar shows 50 μm. (B) The quantitative analysis of NICD-positive cells in WT- and SMA iPSC culture. NICD-positive cells were increased in SMA-iPSC culture. Values represent the mean ± SEM. (n = 3 or 4). ## p < 0.01 and # p < 0.05 versus WT-iPSC MNs (Student's *t*-test). (C-F) NICD expression in whole brain and gastrocnemius of WT (A and B) and SMNΔ7 (C and D) mice which are affected at PND11. Values represent the mean ± SEM. (n = 5 or 6). The cropped blots are used in this Figure and full-length blots are presented in Supplemental Figure 5.

Antigen	Dilution	Application	Catalog #	Isotype	Manufacturer
Oct3/4	1:250	ICC	AF1759	Goat IgG	R&D systems
Sox2	1:250	ICC	AB5603	Rabbit IgG	Millipore
Nanog	1:250	ICC	AF1997	Goat IgG	R&D systems
SSEA4	1:250	ICC	MAB1435	Mouse IgG	R&D systems
TRA1-60	1:250	ICC	MAB4360	Mouse IgG	Millipore
TRA1-81	1:250	ICC	MAB4381	Mouse IgG	Millipore
SSEA1	1:250	ICC	MAB2155	Mouse IgM	R&D systems
Tuj1	1:1000	ICC	MRB-435P	Rabbit IgG	BioLegend
Sox17	1:250	ICC	AF1924	Goat IgG	R&D systems
Vimentin	1:250	ICC	sc-6260	Mouse IgG	Santa Cruz
NICD	1:1000	WB	4147	Rabbit mAb	Cell Signaling
Notch1	1:50	IHC/ICC	sc-6014	Goat IgG	Santa Cruz
Nestin	1:200	IHC/ICC	MAB5326	Mouse IgG	Millipore
GFAP	1:1000	WB	3670	Mouse IgG	Cell Signaling
	1:300	IHC/ICC	3670	Mouse IgG	Cell Signaling
p-STAT3	1:1000	WB	9145	Rabbit IgG	Cell Signaling
	1:50	IHC			
β-actin	1:400	WB	A4700	Mouse IgG	SIGMA
S100	1:1000	WB	ab868	Rabbit IgG	Abcam
	1:300	IHC/ICC			
IL-1β	1:500	WB	ab9722	Rabbit IgG	Abcam
	1:100	IHC/ICC			
Ki67	1:100	IHC	2494306	Rabbit IgG	Millipore
Sox9	1:50	ICC	sc-166505	Rabbit IgG	Santa Cruz

Supplemental Figure 10. Primary antibodies used for immunocytochemistry and western blotting.
